# Tracking of pigment accumulation and secretion in extractive fermentation of *Monascus anka* GIM 3.592

**DOI:** 10.1186/s12934-017-0786-6

**Published:** 2017-10-04

**Authors:** Gong Chen, Qi Bei, Tao Huang, Zhenqiang Wu

**Affiliations:** School of Biology and Biological Engineering, Guangdong Provincial Key Laboratory of Fermentation and Enzyme Engineering, South China University of Technology, Guangzhou, 510006 China

**Keywords:** *Monascus anka*, Intracellular pigment localization, Trans-membrane secretion, Oxidation–reduction, Extractive fermentation

## Abstract

**Background:**

*Monascus* pigments are promising sources for food and medicine due to their natural food-coloring functions and pharmaceutical values. The innovative technology of extractive fermentation is used to promote pigment productivity, but reports of pigment trans-membrane secretion mechanism are rare. In this study, tracking of pigment accumulation and secretion in extractive fermentation of *Monascus anka* GIM 3.592 was investigated.

**Results:**

The increased vacuole size in mycelia correlated with fluorescence intensity (*r* > 0.85, *p* < 0.05), which indicates that intracellular pigments with strong fluorescence accumulated in the cytoplasmic vacuole. After adding nonionic surfactant Triton X-100, the uptake of rhodamine123 (Rh123) and 1-*N*-phenylnaphthylamine (NPN) and the release of K^+^ and Na^+^ rapidly increased, demonstrating that the physiological performances of the cell membrane varied upon damaging the integrity, increasing the permeability, and changing the potential. Simultaneously, the fatty acid composition also varied, which caused a weak fluidity in the membrane lipids. Therefore, the intracellular pigments embedded in Triton X-100 were secreted through the ion channels of the cell membrane. Dense, spherical pigment-surfactant micelles with an average size of 21 nm were distributed uniformly in the extraction broth. Based on the different pigment components between extractive fermentation and batch fermentation, a threefold decrease in the NAD^+^/NADH ratio in mycelia and a more than 200-fold increase in glucose-6-phosphate dehydrogenase (G6PDH) activity in extracellular broth occurred, further suggesting that a reduction reaction for pigment conversion from orange pigments to yellow pigments occurred in non-aqueous phase solution.

**Conclusions:**

A putative model was established to track the localization of *Monascus* pigment accumulation and its trans-membrane secretion in extractive fermentation. This finding provides a theoretical explanation for microbial extractive fermentation of *Monascus* pigments, as well as other non-water-soluble products.

**Electronic supplementary material:**

The online version of this article (doi:10.1186/s12934-017-0786-6) contains supplementary material, which is available to authorized users.

## Background

*Monascus* pigments are a group of mixed azaphilones composed of three color (yellow, orange, and red) components [1]. As functional secondary metabolites, *Monascus* pigments have been widely researched and used as promising pigment additives in the food and pharmaceutical industries [2].

Pigment biosynthesis in *Monascus* spp. is believed to consist of polyketide and fatty acid metabolism [3, 4]. Genomics, transcriptomics, and proteomics analyses have been used to understand pigment biosynthesis and regulatory mechanisms [5, 6]. However, the detailed pathways and enzymes involved in pigment biosynthesis remains unclear or controversial [7]. In submerged fermentation, *Monascus* pigments are mainly biosynthesized and accumulated in the mycelia [8, 9], while the localization of intracellular pigments has not been reported yet. Meanwhile, it is challenging to achieve high intracellular pigment productivity inside the mycelia due to feedback inhibition and production degradation [10, 11].

Extractive fermentation technology is applied as an innovative method for promoting the productivity of fungal intracellular products [12, 13]. It is known as “milking processing”, which describes microbial fermentation of intracellular product in a water–nonaqueous solvent system. With the addition of extractive agent into the fermentation broth, the permeability of cell membrane is enhanced and facilitated the secretion of intracellular product to extracellular broth, and then consecutive extracted the product into the nonaqueous solvent phase [10]. Surfactant in an aqueous solution forms a micelle pseudophase at the surfactant concentration above its critical micelle concentration (CMC). The surfactant micelle aqueous solution can be separated into two phases in a certain temperature, where one is a dilute phase (aqueous solution) and the other is a coacervate phase (surfactant-rich phase). The two-phase system is known as a cloud point system and the temperature is defined as cloud point [14]. Under the cloud point, surfactant can be inter-soluble with water to form a micelle with the hydrophilic side outward and the hydrophobic side inward.

The benefits of exporting the intracellular pigments into extracellular broth via extractive fermentation in Triton X-100 micelle aqueous solution have been investigated experimentally [10, 15]. The surfactant Triton X-100 shows good biocompatibility for cell growth and the hydrophobic pigments can cross the cellular membrane by being “milked” in the artificial nonionic micelle aqueous solution to prevent feed-back inhibition and facilitate pigment production [14, 16]. The “milked” pigments can be concentrated within the surfactant-rich phase (so called coacervate phase) in a cloud-point system induced by a certain temperature level, which provides good feasibility to an efficient downstream separation process [17, 18]. Further study indicated that the cell membrane lipid layer is modified by nonionic surfactant [19] and the pigment conversion occurs during the extraction process [20]. However, the mechanism for pigment trans-membrane secretion in extractive fermentation is unclear and has not yet been reported.

In this study, we investigated the localization of pigment accumulation and its trans-membrane secretion in *Monascus anka* GIM 3.592 extractive fermentation. The image analysis of intracellular pigments, cell membrane physiological characteristics assays, pigment-surfactant micelles observation, NAD^+^/NADH and enzymatic analysis was performed. The response of the putative localization and trans-membrane secretion model of *Monascus* pigments in extractive fermentation was established accordingly.

## Results

### Correlation between intracellular pigments and lipids accumulation

**Fig. 1:**
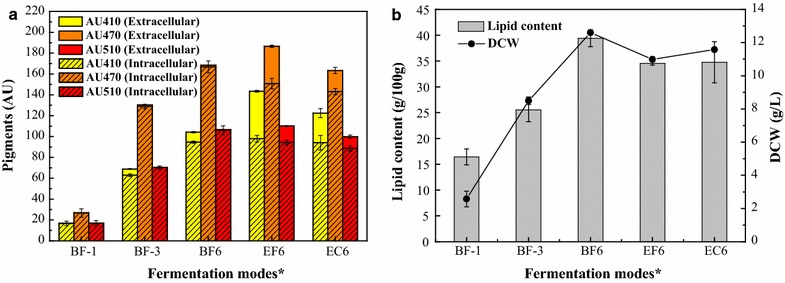
Pigment and lipid synthesis in different fermentation modes. **a** intracellular and extracellular pigment yields; **b** lipid content and DCW. *BF-1 batch fermentation for 1 day, *BF-3* batch fermentation for 3 days, *BF6* batch fermentation for 6 days, *EF6* extractive fermentation for 6 days, *EC6* 6-day batch fermentation broth was added to 40 g/L Triton X-100 for extractive cultivation for 1 h. Values were reported as the mean values ± standard deviations (n = 3)

During batch fermentation, little total (intracellular plus extracellular) pigments were synthesized in the first day with a yield of 30 AU_470_ approximately. Then, pigment production increased and reached approximately 130 AU_470_ on 3rd day and 170 AU_470_ on 6th day (Fig. 1a), with quick cells growth up to 9 g/L and 13 g/L DCW, respectively (Fig. 1b). The lipid content correlated with pigment yield (*r* > 0.90, *p* < 0.05), which was coupled with cell growth. However, the increased lipid synthesis rate was obviously higher than pigmentation in the later stage (Fig. 1b), indicating that the feedback inhibition of intracellular pigments facilitated lipid synthesis. A small amount of intracellular pigments and lipids could be extracted to the extracellular broth simultaneously when 40 g/L Triton X-100 was added to the 6-days of batch fermentation broth for 1 h of extractive cultivation (Fig. 1). Meanwhile, the total pigment yield was unchanged, showing that Triton X-100 was able to facilitate intracellular pigment secretion but was limited by the saturation concentration [20]. A higher yield of both the total pigment and extracellular pigment were obtained with lower lipid content in extractive fermentation compared to batch fermentation (Fig. 1). This result demonstrates that the feedback inhibition of intracellular pigments was relieved and that extractive fermentation facilitated the metabolic channel shift from lipid accumulation to pigment yield [21].

### Intracellular pigment localization during fermentation

**Fig. 2:**
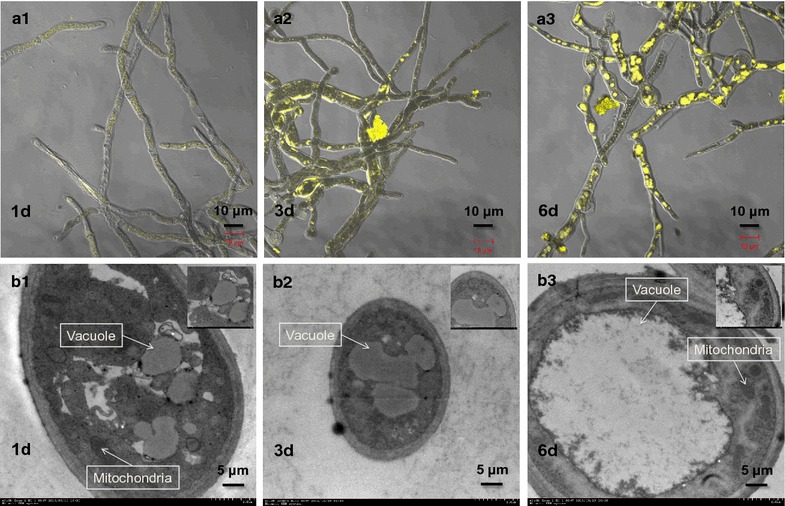
*Monascus* mycelium image by LSCM and TEM in different growth phase. **a1**–**a3** Pigment fluorescence and mycelium morphology by LSCM at 1st, 3rd and 6th day of batch fermentation; **b1**–**b3** internal structure and organelles of the mycelium by TEM at 1st, 3rd and 6th day of batch fermentation

LSCM micrography showed that the mycelia were very thin and long with little fluorescence on the first day of batch fermentation (Fig. 2a1). Three days later, the mycelia became shorter and branched, and the fluorescence intensity also increased (Fig. 2a2). By the end of fermentation, all the mycelia were nearly full of fluorescence (Fig. 2a3). The fluorescence intensity was constantly increased along with the fermentation time (Table 1), which was highly correlated with the intracellular pigment yields (AU_410_, *r* > 0.90, *p* < 0.05; AU_470_, *r* > 0.95, *p* < 0.01; AU_510_, *r* > 0.90, *p* < 0.05). As shown in Fig. 2b, during batch fermentation, the cytoplasmic vacuole size imaged by TEM also increased with the fermentation time in a manner consistent with fluorescence intensity (*r* > 0.85, *p* < 0.05). The vacuoles occupied a considerable area in the cytoplasm and were irregularly scattered, which was similar to the different areas of fluorescence pigment in mycelia (Fig. 2a). The vacuoles may act as reservoirs of the intracellular *Monascus* pigment, since they are less homogeneous in shape and larger than lipid droplets.

**Table 1:** Pigment fluorescence variance in *Monascus* mycelia through different growth phases

Fermentation time (days)	Fluorescent intensity (%)
1	1.67 ± 0.01a
3	6.47 ± 0.02b
6	8.31 ± 0.02c

The data are expressed as the mean values ± standard deviations (n > 3). Mean values in a column with different lowercase letters (a, b, c) are significantly different (*p* < 0.05)

### Cell membrane changes during extractive fermentation

**Fig. 3:**
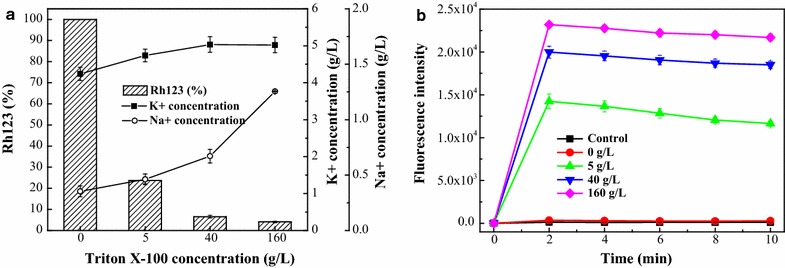
Variation in **a** K^+^ and Na^+^ concentration, Rh123 relative content, and **b** NPN fluorescent intensity in extractive cultivation with different Triton X-100 concentrations

The study findings indicated that the K^+^ and Na^+^ concentrations in the extracellular broth increased when Triton X-100 was added to the batch fermentation broth (Fig. 3a). This increasing trend became more obvious with higher Triton X-100 concentrations, which indicated that K^+^ and Na^+^ release upon the addition of Triton X-100 was concentration-dependent. Meanwhile, the fluorescence intensity of Rh123 significantly decreased from 100 to 24% during extractive cultivation with 5 g/L of Triton X-100. The intensity continuously declined to 7 and 4% when the Triton X-100 concentration was increased to 40 and 160 g/L, respectively (Fig. 3a). Additionally, there was rapid NPN uptake as soon as Triton X-100 mixed with *Monascus* fungi suspensions, with the maximum achieved in approximately 2 min. Thereafter the NPN uptake was almost unchanged or even declined until 10 min (Fig. 3b). The fluorescence increase was dose-dependent so that the maximum fluorescence was greater with higher Triton X-100 concentrations, which was in accordance with the membrane integrity and potential results. In control suspensions (1% HAc or 0 g/L Triton X-100), there was almost no NPN uptake after 10 min. These results indicated that, during the extractive fermentation, the physiological performances of the cell membrane were varied by damaging the integrity, increasing the permeability, and changing the potential.

The fatty acid composition of the cell membrane in different fermentations is shown in Table 2. The major fatty acids produced by batch fermentation were tetradecanoic acid (C14:0), palmitic acid (C16:0), heptadecanoate (17:0), stearic acid (C18:0), eicosanoic acid (20:0), hexadecenoic acid (16:1), oleic acid (C18:1), linoleic acid (C18:2), and linolenic acid (C18:3). Though the fatty acid composition did not varied, the unsaturated/saturated fatty acid ratio and the index of unsaturated fatty acid (IUFA) value decreased significantly (*p* < 0.05) from 2.53 and 101.36 in batch fermentation to 2.18 and 97.15 in extractive cultivation. Notably, both the major fatty acid composition and IUFA value declined in extractive fermentation (Table 2). This finding suggests that Triton X-100 reduced the fluidity of *Monascus anka* GIM 3.592 membrane lipids.

**Table 2:** Cell membrane fatty acid composition (% total fatty acid) in *Monascus anka* with different fermentation modes

Fatty acid composition	Fermentation modes^a^		
	BF	EC	EF
Saturated fatty acid			
Tetradecanoate (14:0)	0.09 ± 0.00	0.17 ± 0.03	–
Palmitic acid (16:0)	15.74 ± 0.02	16.20 ± 0.10	20.11 ± 1.01
Heptadecanoate (17:0)	0.18 ± 0.02	0.35 ± 0.00	–
Stearic acid (18:0)	12.83 ± 0.65	14.50 ± 0.66	14.20 ± 0.54
Eicosanoic acid (20:0)	0.09 ± 0.00	0.21 ± 0.00	–
Unsaturated fatty acid			
Hexadecenoic acid (16:1)	0.16 ± 0.02	0.28 ± 0.05	–
Oleic acid (18:1)	42.57 ± 2.33	41.62 ± 0.06	38.87 ± 0.95
Linoleic acid (18:2)	26.37 ± 1.08	24.76 ± 0.04	25.16 ± 0.81
Linolenic acid (18:3)	1.97 ± 0.05	1.91 ± 0.03	1.66 ± 0.01
Unsaturated/saturated fatty acid ratio^b^	2.45 ± 0.30a	2.18 ± 0.23b	1.91 ± 0.11c
IUFA (index of unsaturated fatty acid)^c^	101.36 ± 0.49a	97.15 ± 0.29b	94.16 ± 0.26c

^a^ *BF* batch fermentation for 6 days, *EF* extractive fermentation for 6 days, *EC* day-6 batch fermentation broth was added to 40 g/L Triton X-100 to conduct extractive cultivation for 1 h. The data are expressed as the mean values ± standard deviations (n = 3). Mean values in a row with different lowercase letters (a, b, c) are significantly different (*p* < 0.05)

^b^(C16:1 + C18:1 + C18:2 + C18:3)/(C14:0 + C16:0 + C18:0 + C20:0)

^c^C16:1 + C18:1 + 2 × C18:2 + 3 × C18:3

### Transformation and colloidization of pigments in extractive fermentation

**Fig. 4:**
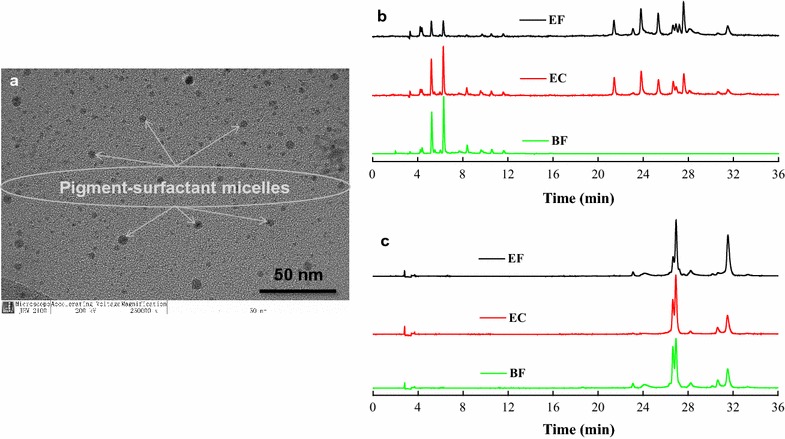
Distribution of pigment-surfactant micelles (**a**) in extracellular broth and HPLC–PDA chromatogram of extracellular (**b**) and intracellular (**c**) pigments fermented under different modes. *BF* batch fermentation for 6 days, *EF* extractive fermentation for 6 days, *EC* 6-day batch fermentation broth was added to 40 g/L Triton X-100 for extractive cultivation for 1 h

As most pigments were hydrophobic and the surfactant was amphipathic in aqueous solutions, Triton X-100 was more inclined to form micelles under the cloud point in which the hydrophobic pigments were embedded to form pigment-surfactant mixed micelles [10]. Therefore, the pigment secretion was limited by the saturation concentrations of Triton X-100 [20]. TEM micrography (Fig. 4a) revealed the occurrence of dense, spherical micelles with an average size of 21 nm, indicating that the pigment-surfactant micelles in which pigments and Triton X-100 coexisted were distributed uniformly in extractive fermentation broth. To further examine the distribution of Triton X-100 between both sides of the cell wall during extractive fermentation, the mature cells were soaked in Triton X-100 (Table 3, initial) aqueous solutions for 1 h extractive cultivation. It found that a high concentration of Triton X-100 that embedded intracellular pigments existed in the extracellular broth (Table 3, 0). Subsequently, the mycelia were collected and dispersed in the same volume of distilled water for 1 h, and then, the mycelia suspension solution was centrifuged again to determine the extracellular Triton X-100 concentration in supernatant (Table 3, 1). After that, the mycelia were collected to repeat the above washing operations with distilled water for 2–7 times, and the supernatants were used to determine the extracellular Triton X-100 concentration in sequence (Table 3, 2–7). After washed seven times, the mycelia were collected to detect the intracellular Triton X-100 concentration (Table 3, cellular). It showed that the concentration of Triton X-100 inside the cell was much higher than that in the extracellular broth even after seven times washing with distilled water (Table 3, 7 and cellular). This finding indicates that Triton X-100 might enter cells and extract pigment back to the extracellular broth, and the concentration of Triton X-100 in aqueous micellar systems might be a rapid dynamic equilibrium process during extractive cultivation.

**Table 3:** Triton X-100 concentration in extracellular and cellular environment after washing the extracted mature cells seven times

Washing time	Initial	Extracellular								Cellular
		0	1	2	3	4	5	6	7	
Triton X-100 concentration (g/L)	38.40 ± 0.41	30.71 ± 1.71	3.01 ± 0.18	1.73 ± 0.23	0.73 ± 0.20	0.39 ± 0.02	0.39 ± 0.01	0.38 ± 0.05	0.14 ± 0.03	0.83 ± 0.05

The data are expressed as the mean values ± standard deviations (n = 3)

The extracellular pigment components in extractive fermentation were different from those in batch fermentation (Fig. 4b), which indicates pigment conversion during the extraction process, as shown in a previous study [20]. Moreover, the high G6PDH activity was observed in extractive cultivation (1.485 U/mL) and was higher in extractive fermentation (2.415 U/mL) than the extremely low level (0.015 U/mL) observed in batch fermentation (Table 4). Additionally, the NAD^+^/NADH ratio in cells decreased when adding Triton X-100 into batch fermentation broth for extractive cultivation, and the decreasing trend became more obvious in extractive fermentation (Table 4). This finding illustrates that the NAD^+^/NADH ratio in the extracellular broth may increase due to the metabolic balance of NADH to NAD^+^ conversion during the trans-membrane secretion process. Therefore, there may be a reduction reaction for pigment conversion during the trans-membrane transport process in extractive fermentation.

**Table 4:** The NAD^+^/NADH and G6PDH activity in different fermentation modes

Fermentation mode^a^	NAD^+^/NADH	G6PDH (U/mL)
BF	4.23 ± 0.10a	0.015 ± 0.002a
EC	2.35 ± 0.21b	1.485 ± 0.023b
EF	1.47 ± 0.28c	2.415 ± 0.026c

^a^ *BF* 6-day batch fermentation, *EF* 6-day extractive fermentation, *EC* 6-day batch fermentation broth was added to 40 g/L Triton X-100 to conduct extractive cultivation for 1 h. The data are expressed as the mean values ± standard deviations (n = 3). Mean values in a column with different lowercase letters (a, b, c) are significantly different (*p* < 0.05)

## Discussions

*Monascus* pigments synthesized by natural strains mainly contain intracellular pigments that accumulate in mycelia [10]. In this study, increasing intercellular mass or granular inclusions in mycelia were observed (Fig. 2a) with the continuous accumulation of hydrophobic intracellular pigments, which was coupled with cell growth in batch fermentation (Fig. 1). Moreover, the fluorescence intensity imaged by LCSM was also constantly increased with the increment of intracellular pigments in *Monascus* mycelia (*r* > 0.90, *p* < 0.05). Some intracellular pigments are found to have strong fluorescence [2], and intercellular mass or granular inclusions have been hypothesized as storage for an accumulation of synthesized intracellular pigments [22]. Interestingly, the size of vacuoles in *Monascus* mycelia increased in conjunction with fluorescence intensity (*r* > 0.85, *p* < 0.05), and occupied a high proportion of cytoplasm during batch fermentation (Fig. 2b). The fungal vacuole is an important cellular organelle in metabolite storage and cytosolic ion homeostasis [23] as well as some key enzymes involved in secondary metabolites [24]. The morphology of vacuoles varies among different species and the size of vacuoles increases during the cell cycle [25]. Moreover, the irregular scattering of vacuoles in the cytoplasm was consistent with the random distribution of pigment fluorescence from the mycelia (Fig. 2). The vacuoles may act as repositories for these intracellular hydrophobic pigments, which is consistent with the results reported by Suh and Shin [26]. Astaxanthin, an analogue compound of *Monascus* pigments, has also been reported being accumulated in yeast cell liposomes (similar to vacuoles) with an irregular, scattered distributed [27]. The lipid content showed a relationship to pigment yield in batch fermentation (*r* > 0.90, *p* < 0.05) and could be extracted to extracellular broth with the intracellular pigments during extractive cultivation (Fig. 1). This finding indicated that lipids might also be located in the vacuole with the intracellular pigments due to a similar precursor acetyl CoA in the metabolic pathway [28] and similar hydrophobic properties [21].

Extractive fermentation with the nonionic surfactant Triton X-100 is an efficient method of promoting *Monascus* pigment production, and some intracellular hydrophobic pigments that are only distributed in mycelia demonstrated transport behavior through the cell membrane to the extracellular broth [14]. Herein, similar results showed that intracellular pigment components accumulated in the cytoplasmic vacuoles could be well extracted to the extracellular environment with extractive fermentation using Triton X-100 (Fig. 4b, c). Moreover, high yields of extracellular and intracellular pigment were obtained, with a little decline of biomass compared with the traditional batch fermentation (Fig. 1). This indicated that the *Monascus* mycelia was grown properly and maintained a high pigment biosynthesis activity, although the cells had been sustained by the toxicity of high Triton X-100 concentration (40 g/L). A previous study found that mycelia morphology consisting of hyphae and mycelial pellets was influenced and damaged in extractive fermentation of *Monascus anka* [22]. It is also reported that the toxicity of Triton X-100 molecules would be inserted in the cell membrane lipid bilayer, and then affected the cell membrane structure [29]. In this study, it was found the composition and physiological performances of the cell membrane were changed to increase the cell membrane permeability in extractive fermentation (Fig. 3; Table 2). Moreover, TEM micrography showed that the cell wall and the cytoplasmic vacuoles of *Monascus* mycelia were destructed with the addition of Triton X-100 in batch fermentation broth, and the internal contents including pigments were irregularly distributed. This also traced by the LSCM that the pigment fluorescence was instantly declined due to the increase of cell membrane permeability to facilitate intracellular pigment secretion (will be published in the next work). The determination of Triton X-100 concentration also showed that the Triton X-100 concentration in cellular was much higher than that in supernatant even after seven times washing the extracted mature cells with distilled water (Table 3). These facts indicated that Triton X-100 might enter the cell and extract pigment back to the extracellular broth.

The cytoplasmic cell membrane is a structural component, which may become damaged and functionally invalid when fungi suspensions are exposed to anti-microbial agents [30]. Organic matter, such as Tween-80, toluene, ether, and chitosan, can increase cell membrane penetrability and cause cytoplasm leakage [31, 32]. In this work, the permeability and integrity of the cell membrane varied significantly via increasing uptake of the hydrophobic probe NPN and release of K^+^ or Na^+^ [30] in extractive cultivation induced by Triton X-100 (Fig. 3). Moreover, the release of K^+^ or Na^+^ also demonstrated an influence on the ion channel as well as cell membrane potential variation [33, 34]. The cell membrane potential is a good indicator for cell vitality and functional characteristics, and changes in the cell membrane potential (Fig. 3a) induce potassium (K^+^) channel opening. The enhancement of K^+^ or Na^+^ secretion leads to further hyperpolarization of the membrane potential, resulting in the variation of other ion channels [33]. Multiple ion channels for Na^+^, K^+^, Ca^+^, and Cl^−^ exist in the cell membrane facilitate material transport [35]. Small particles, such as inorganic ions and small organic molecules, can leave the cell via specialized trans-membrane carrier or channel proteins [13]. Therefore, due to their low molecule weights, intracellular pigments were likely to be taken up and excreted through trans-membrane transport from ion channels in the cell membrane, because the decreased ratio of unsaturated/saturated fatty acid in *Monascus anka* (Table 2) resulted in reduced membrane lipid fluidity [36].

Characteristic variation of intracellular and extracellular pigments has been found in fed-batch and continuous extractive fermentation with *Monascus anka* [37]. In this work, many new extracellular pigments were found in extractive fermentation, which were different from the components biosynthesized in batch fermentation (Fig. 4b, c). The generated pigments possess characteristic spectra of yellow pigments with the peak absorbance at approximately 430 nm and four new pigment components have separated in our late work [38]. It has been reported that the intracellular yellow and orange pigments are converted due to the enzyme catalytic reaction in the non-aqueous phase solution during the trans-membrane secretion process [20]. Herein, it was showed that the NAD^+^/NADH ratio in mycelia was decreased threefold, indicating a conversion from NADH to NAD^+^ in extracellular broth during extractive fermentation of *Monascus anka* (Table 4). The NAD^+^/NADH ratio reflects the intracellular oxidation–reduction capacity [39]. Some pathways in cell growth and production metabolism can be controlled by maintaining the redox level balance via adjusting the NADP^+^/NADPH and NAD^+^/NADH ratios [40]. Moreover, high G6PDH activity was found in extractive broth compared to the extremely low level in batch fermentation (Table 4). These results were consistent with the fact that the orange pigment can be transformed into yellow pigments by chemical hydrogenation with the help of related enzymes [4, 41]. Additionally, the oxidation–reduction potential (ORP) value varied with the increase in Triton X-100 concentration (Additional file 1: Table S1). The ORP reflects the redox level for fermentation broth has been studied as a control parameter in fermentation processes [42], and may be a novel indicator for *Monascus* yellow pigment biosynthesis [43]. Therefore, there must be a reduction reaction for orange pigment to yellow pigment during the trans-membrane transport process in extractive fermentation.

**Fig. 5:**
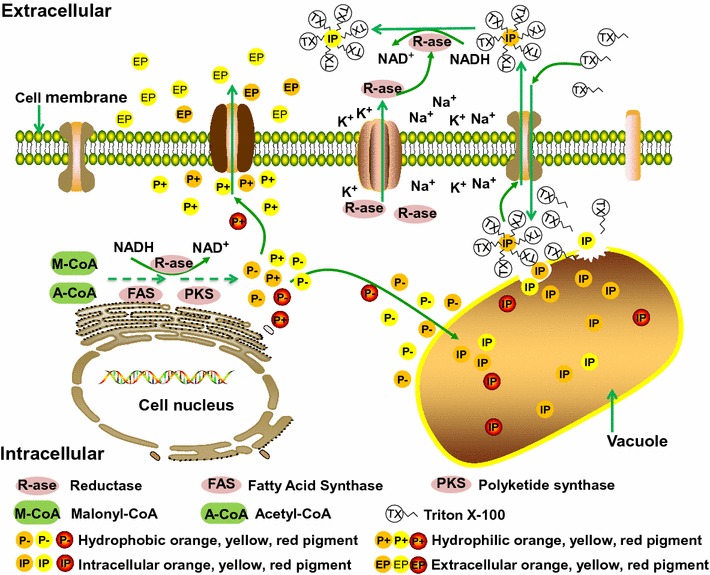
Putative model of *Monascus* pigment accumulation and trans-membrane secretion in extractive fermentation

In conclusion, the results of this work indicate that Triton X-100 damaged the cell wall and increased the cell membrane permeability. Afterwards, Triton X-100 entered into the cell and embedded intracellular pigments that accumulated in the cytoplasmic vacuoles for secretion through the cell membrane ion channels. The coexisting pigment-surfactant micelles were distributed uniformly with dense, spherical micelles in the extracellular broth. Furthermore, orange pigment in the extracellular broth could be converted to yellow pigment through a reduction reaction. Based on these facts, we established a putative localization model of *Monascus* pigment accumulation and its trans-membrane secretion in extractive fermentation (Fig. 5). In this model, pigment secretion occurs through rapid trans-membrane transport (data not shown), which is limited by the saturation concentrations of Triton X-100 [20]. The mechanism discovered in this manuscript provides a theoretical explanation for microbial extractive fermentation of *Monascus* pigments as well as other non-water-soluble products.

## Methods

### Microorganism and fermentation media

The *Monascus anka* GIM 3.592 fungi was deposited in Guangdong culture collection center of microbiology (GDMCC/GIMCC, China) and maintained on potato dextrose agar (PDA) medium at 4 °C.

The seed medium consisted of glucose 20 g, yeast extract 3 g, peptone 10 g, KH_2_PO_4_ 4 g, KCl 0.5 g, and FeSO_4_·7H_2_O 0.01 g/L of distilled water. The fermentation medium consisted of glucose 50 g, (NH_4_)_2_SO_4_ 5 g, KH_2_PO_4_ 5 g, MgSO_4_·7H_2_O 0.5 g, KCl 0.5 g, MnSO_4_·H_2_O 0.03 g, ZnSO_4_·7H_2_O 0.01 g and FeSO_4_·7H_2_O 0.01 g/L of distilled water. The initial pH in both the seed and fermentation media was not controlled.

### Batch fermentation and extractive batch fermentation

A *Monascus anka* GIM 3.592 sub-culture was maintained on a PDA plate at 30 °C for 7 days to collect spore suspensions by adding 6 mL of 0.1% (m/v) Tween-80 solution onto each plate. Then 3 mL of spore suspension was inoculated into a 250-mL Erlenmeyer flask containing 50 mL of the seed medium and incubated at 30 °C for 30 h in a rotary shaker at 180 rpm. Afterwards, 2 mL of the seed culture broth was withdrawn and inoculated into 25 mL of fermentation medium in 250-mL Erlenmeyer flasks to continue the submerged fermentation. Cultivation was performed at 30 °C and 180 rpm for 6 days as batch fermentation (BF). Extractive fermentation (EF) was performed in the same manner as the batch fermentation except that both 2 mL of the seed culture broth and 40 g/L Triton X-100 were added into 25 mL of fermentation medium in 250-mL Erlenmeyer flasks.

### Pigment, biomass and lipid assays

The estimation of pigment concentration and biomass followed the same method as detailed in our previous work [11, 43]. It has been reported that the fermented *Monascus* pigments are mixtures containing different types of individual pigments [1, 2, 9]. Due to the high complexity in the pigment compositions, it is difficult to quantify the composition of each chemical compound in grams or moles. Alternatively, *Monascus* pigment content is generally demonstrated by their integrated color characteristics through the visible spectrum and the visible absorbance at 410 nm for yellow pigments, 470 nm for orange pigments and 510 nm for red pigments [4, 8, 10, 11, 14–16, 19, 21, 26, 36, 43–46]. Additionally, the total pigment yield was defined as the yields of intracellular pigments plus extracellular pigments. The absorbance analysis of extracellular pigments was directly determined by supernatant of fermentation broth in the water-solvent two-phase system according to the common method [10, 11, 14–16, 19–22, 37, 38]. The biomass characteristic to dry cell weight (DCW) was determined by drying the mycelia at 60 °C until a constant weight was achieved. Additionally, the estimation of lipid content followed the same method as described by Huang et al. [36].

### Image analysis of intracellular pigment localization by LSCM and TEM

#### Laser scanning confocal microscopy (LSCM)

Image analysis of intracellular pigments by LSCM was performed as follows: 1 mL of batch fermentation broth withdrawn from the cultivation for the 1st, 3rd, and 6th day, respectively, were centrifuged at 8000 rpm for 5 min. The mycelia were collected and washed three times with distilled water. Afterwards, the mycelia were re-suspended with 0.1 M phosphate buffer solution (PBS) and imaged using LSCM (LSM 710, Zeiss, Germany) with an excitation wavelength of 488 nm and an emission wavelength of 542–573 nm. In particular, the fluorescent image of intracellular pigment was excited by itself without addition of fluorescent reagent.

#### Transmission electron microscope (TEM)

Mycelia were prepared similar to the LSCM method. The washed mycelia by distilled water were then fixed with 1 mL of fixative (4% glutaraldehyde and 3% paraformaldehyde) for 4 h, collected by centrifugation at 8000 rpm for 5 min and washed three times with 0.1 M PBS to remove residual fixative. The mycelia were then fixed with 0.1 mL of 1% osmic acid overnight, washed four times with 0.1 M PBS, and dehydrated successively with 30, 50, 70, 85, 95, and 100% (v/v) ethanol. Subsequently, the mycelia were embedded in resin and further polymerized at 65 °C for 2 days. Finally, the treated samples were sliced using an ultra-microtome (UCT, Leica, Germany), stained with uranium acetate and lead citrate, and observed using TEM (H-600, Hitachi, Japan).

### Physiological performances of cell membrane assays

#### GC–MS analysis of cell membrane fatty acids

After cultivation for 6 days, the batch fermentation broth was added to 40 g/L Triton X-100 for extractive cultivation (EC) at 30 °C with 180 rpm for 1 h. Then, 25 mL of broth was withdrawn from batch fermentation (BF), extractive cultivation (EC), and extractive fermentation (EF), respectively, to collect mycelia to extract, purify and methylate the cell membrane fatty acids according to the method described by Wang et al. [19]. The fatty acid composition was analyzed using the gas chromatography-mass spectrometry (GC–MS) method described by Huang et al. [36].

#### K^+^ and Na^+^ concentration analysis using FAAS

After cultivation for 6 days, the batch fermentation broth was withdrawn and 0, 5, 40, and 160 g/L Triton X-100, respectively, were added, followed by incubation at 30 °C with 180 rpm for 1 h. Subsequently, the extractive cultivation broths were centrifuged at 8000 rpm for 5 min to separate the mycelia, and the supernatants were used to determine the K^+^ and Na^+^ concentration using a flame atomic absorption spectrometry (FAAS) method described by Wei et al. [47].

#### Determination of cell membrane potential

Cell membrane potential was determined using the rhodamine123 (Rh123) assay [48]. The batch fermentation broth cultivated for 6 days was withdrawn and centrifuged at 8000 rpm for 5 min. The collected wet mycelia were washed three times and re-suspended with 0.1 M PBS. The suspension broth was then diluted 10-times. Afterwards, 1 mL of diluted suspension broth was mixed with 1.5 mL of 0, 5, 40, and 160 g/L Triton X-100 aqueous solutions, respectively, followed by extractive cultivation at 30 °C for 30 min. Subsequently, 10 μL of 1 g/L Rh123 was added into the extractive cultivation broths for 10 min incubation in the dark. Finally, the reaction broths were centrifuged at 8000 rpm for 5 min to remove the supernatants, and the mycelia were washed three times and re-suspended with 0.1 M PBS to determine the florescence intensity using a fluorescence spectrophotometer (Spectra Max M5, USA) with an excitation wavelength of 488 nm and an emission wavelength of 530 nm.

#### Determination of outer membrane permeabilization

Triton X-100 outer membrane (OM) permeabilization activity was analyzed using a 1-*N*-phenylnaphthylamine (NPN) assay [49] according to the method described by Xing et al. [29] with some modification. The batch fermentation broth cultivated for 6 days was withdrawn and centrifuged at 8000 rpm for 5 min. The collected wet mycelia were washed three times and re-suspended with 0.5% NaCl solution, and the suspension broth was then diluted 100-times. The solutions of 0, 5, 40, and 160 g/L Triton X-100 as well as 1% acetic acid solution (control) were adjusted to pH 4.0. Then, 1.5 mL of Triton X-100 solutions or acetic acid solution was mixed with 20 μL of 1 mM NPN. Afterwards, 1 mL of diluted cell suspension was added and the fluorescence was recorded immediately as a function of time due to partitioning of NPN into the OM. The fluorescence was recorded with a fluorescence spectrophotometer (Spectra Max M5, USA) with an excitation wavelength of 350 nm and an emission wavelength of 420 nm, respectively.

### NAD^+^/NADH and reductase analysis

Twenty-five milliliter of broths from BF, EC and EF, respectively, were withdrawn and centrifuged at 8000 rpm for 5 min to separate the mycelia. The supernatants were used to determine the reductase by the glucose-6-phosphate dehydrogenase (G6PDH) assay according to the method described by Liao et al. [50] with some modifications. The reaction mixture (3 mL) with 0.6 mL of 1 M Tris–HCl (pH 8.0), 2.1 mL of distilled water, 0.15 mL of 0.1 M glucose-6-phosphate disodium, 0.05 mL of 0.1 M NADP^+^, and 0.1 mL of the crude enzyme solution (supernatants) was added to catalyze the reduction reaction at 30 °C for 20 min. The G6PDH activity was determined using an ultraviolet spectrophotometer (UV-2802S, Unico, USA) at 340 nm. One unit of enzyme activity was defined as per milliliter of fermentation broth that dropped 1.0 of A_340nm_ per min under the assay conditions. The results were expressed as U/mL.

Meanwhile, the mycelia were collected to extract the NAD^+^ and NADH for high-performance liquid chromatography (HPLC) determination according to the method described by Liu et al. [51] with some modifications. To extract the NADH, the mycelia were grinded by liquid nitrogen and then transferred to 10 mL of 0.4 M KOH. After 10 min of cultivation in a 30 °C water bath, the supernatants were collected by centrifugation at 8000 rpm for 10 min and neutralized to pH 7.0 with 0.1 M HCl. For NAD^+^, the grinded mycelia were collected and soaked in 10 mL of HCl (pH 1.3). After 10 min of cultivation in a 50 °C water bath, the supernatants were collected by centrifugation at 8000 rpm for 10 min and neutralized to pH 7.0 with 0.1 M KOH. The HPLC system (e2695, Waters, USA) was equipped with a 2998 Photodiode Array (PDA) detector (2998, Waters, USA) and a Zorbax *Ecipse Plus* C18 column (5 μm, 250 × 4.6 mm, Waters, USA). The mobile phase consisted of 95% eluent A (0.01 M KH_2_PO_4_) and 5% eluent B (methanol) at a flow rate of 0.800 mL/min. The detection temperature of the column oven was set at 30 °C and the detection wavelength was set to 260 nm.

### Pigment-surfactant micelle distribution analysis

Micrographs of the pigment-surfactant micelles were recorded on a TEM (JEM-2100, JEOL, Japan) at 200 kV. The supernatant of the extractive fermentation broth with definite concentration of pigments and Triton X-100 (approximately 40 g/L for Triton X-100 and 30 AU_470_ for pigments) were prepared and then a drop of the solutions was dispersed on the surface of a TEM copper grid (200 meshes). The solution was dried before data acquisition and at least three different areas were scanned for each sample.

The nonionic surfactant Triton X-100 concentration was determined using the HPLC method. Methanol was used as the eluent with a flow rate of 1.000 mL/min. The detection temperature of the column oven was set to 30 °C and the detection wavelength was set to 277 nm. The pigment compositions were analyzed using HPLC method according to our previous work [20]. The mobile phase consisted of eluent A (water: phosphoric acid = 10,000:3, v/v) and eluent B (acetonitrile) at a flow rate of 1.000 mL/min, and the elution gradient was as follows: 0 min, 80% A; 25 min, 20% A; 35 min, 20% A; 36 min, 80% A; and 40 min, 80% A. The temperature of the column oven was set to 30 °C and the detection wavelength was set to 410 nm.

### Statistical analysis

Data were expressed as the mean values ± standard deviation (SD) for each measurement. The data were subjected to ANOVA analysis and significance of differences was determined by Duncan’s multiple range tests where necessary. *p* < 0.05 was considered statistically significant in all cases. All analyses were performed with the SPSS software package (version 22.0, SPSS Inc., Chicago, IL, USA).

## Additional file

**Additional file 1: Table S1.** ORP of intracellular and extracellular in extractive cultivation of* Monascus anka* with different Triton X-100 concentrations.
