# Supplementary material for: Tracking of pigment accumulation and secretion in extractive fermentation of Monascus anka GIM 3.592
Source: Microb Cell Fact. 2017 Oct 4;16:172. doi: 10.1186/s12934-017-0786-6 (PMC5628469; doi:10.1186/s12934-017-0786-6)
Supplement: Supplementary file 1 — Additional file 1: Table S1. ORP of intracellular and extracellular in extractive cultivation of Monascus anka with different Triton X-100 concentrations. [file 12934_2017_786_MOESM1_ESM.docx]

**Additional file 1**

**Table S1.** ORP of intracellular and extracellular in extractive cultivation of *Monascus* *anka* with different Triton X-100 concentrations

| Triton X-100 concentration (g/L) | ORP (mV) | |
| --- | --- | --- |
|  | Extracellular broth | Intracellular broth |
| 0 | 233.3±5.94a | 215.4±7.63a |
| 5 | 236.5±1.84a | 247.1±2.76b |
| 40 | 242.7±1.63a | 250.6±0.85b |
| 160 | 275.7±2.83b | 249.3±0.71b |

* The data are expressed as the mean values ± standard deviation (n=3). Mean values in a column with different lowercase letters (a, b) are significantly different (*p*<0.05).
